# Supplementary material for: Identification of photoperiod-induced specific miRNAs in the adrenal glands of Sunite sheep (Ovis aries)
Source: Front Vet Sci. 2022 Jul 22;9:888207. doi: 10.3389/fvets.2022.888207 (PMC9354845; doi:10.3389/fvets.2022.888207)
Supplement: Supplementary file 1 [file Data_Sheet_1.ZIP › Supplementary file/miRDeep2 file.zip!/miRDeep2 file/Novel_Structure/SPLP42A3/Novel_178.pdf]

Provisional ID	:	NC_019484.2_39347
Score total	:	7.2
Score for star read(s)	:	3.9
Score for read counts	:	0.1
Score for mfe	:	1.6
Score for randfold	:	1.6
Score for cons. seed	:	
Total read count	:	12
Mature read count	:	7
Loop read count	:	0
Star read count	:	5

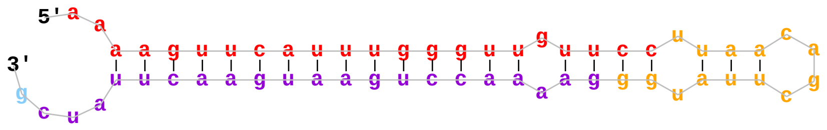

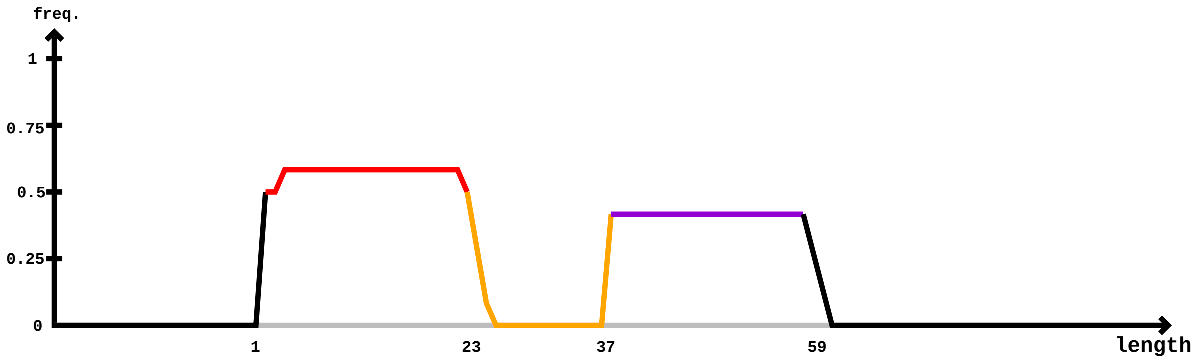

Mature Star

```
5'- uacuacuauuguguuggccaaaaaguucauuuggguuguuccuuaacagcuuaugggaaaaccugaaugaacuuaucggucaacccaauacguuagagaaauguagagugacag   -3'    obs
    uacuacuauuguguuggccaaaaaguucauuuggguuguuccuuaacagcuuaugggaaaaccugaaugaacuuaucggucaacccaauacguuagagaaauguagagugacag          exp
    ..(((((((((.(((((((...(((((((((((((((.((((.(((....))).)))).)))))))))))))))...))))))).))))).).)))..................    reads      mm        sample
    ....................aaaaguucauuuggguuguuc.........................................................................    1          0         seq
    ....................aaaaguucauuuggguuguucc........................................................................    3          0         seq
    ....................aaaaguucauuuggguuguuccA.......................................................................    1          1         seq
    ....................aaaaguucauuuggguuguuccuu......................................................................    1          0         seq
    ......................aaguucauuuggguuUuucc........................................................................    1          1         seq
    ........................................................gaaaaccugaaugaacuuauc.....................................    3          0         seq
    ........................................................gaaaaccugaaugaacuuaucg....................................    2          0         seq
```
